# A Scientometric Analysis and Visualization of Prosthetic Foot Research Work: 2000 to 2022

**DOI:** 10.3390/bioengineering10101138

**Published:** 2023-09-28

**Authors:** Qiu-Qiong Shi, Kit-Lun Yick, Jinlong Wu, Xujia Huang, Chi-Yung Tse, Mei-Ki Chan

**Affiliations:** 1 Laboratory for Artificial Intelligence in Design, Hong Kong, China; gaby-qiuqiong.shi@polyu.edu.hk; 2 School of Fashion and Textiles, The Hong Kong Polytechnic University, Hong Kong, China; meiki-mickey.chan@polyu.edu.hk; 3 College of Physical Education, Southwest University, Chongqing 400715, China; 1800371011@email.szu.edu.cn; 4 School of Recreational Sports and Tourism, Beijing Sport University, Beijing 100084, China; stefan0207@163.com; 5 Centre for Orthopaedic Surgery, Hong Kong, China; oandp@cos.hk

**Keywords:** scientometric analysis, bibliometric analysis, visualization, prosthetic foot, lower-extremity amputation

## Abstract

This study aims to highlight recent research work on topics around prosthetic feet through a scientometric analysis and historical review. The most cited publications from the Clarivate Analytics Web of Science Core Collection database were identified and analyzed from 1 January 2000 to 31 October 2022. Original articles, reviews with full manuscripts, conference proceedings, early access documents, and meeting abstracts were included. A scientometric visualization analysis of the bibliometric information related to the publications, including the countries, institutions, journals, references, and keywords, was conducted. A total of 1827 publications met the search criteria in this study. The related publications grouped by year show an overall trend of increase during the two decades from 2000 to 2022. The United States is ranked first in terms of overall influence in this field (*n* = 774). The Northwestern University has published the most papers on prosthetic feet (*n* = 84). *Prosthetics and Orthotics International* has published the largest number of studies on prosthetic feet (*n* = 151). During recent years, a number of studies with citation bursts and burst keywords (e.g., diabetes, gait, pain, and sensor) have provided clues on the hotspots of prosthetic feet and prosthetic foot trends. The findings of this study are based on a comprehensive analysis of the literature and highlight the research topics on prosthetic feet that have been primarily explored. The data provide guidance to clinicians and researchers to further studies in this field.

## 1. Introduction

Around 57.7 million people worldwide underwent limb amputation in 2017, with Asia having the highest number of amputations, followed by Western Europe, North Africa, the Middle East, and then North America [1]. Approximately 150,000 patients undergo a lower extremity amputation (LEA) in the United States each year [2], and the number of people who will be living with an amputation in the United States is estimated to be approximately 3.6 million by 2050 [3,4]. In China, 1.58 million Chinese people experienced lower limb loss in 2006, and the number of Chinese people who will be living with an amputation is estimated to reach 2.33 million in 2030 [5]. However, since walking is an essential part of daily life, those with an LEA suffer from significant loss of mobility, which is inconvenient and causes hardship [4,6]. Prosthesis use can disguise the partial or complete loss of a lower limb in appearance, while assisting those with an LEA to walk or participate in daily activities and enjoy a certain amount of physical movement and ability to self-care [7,8,9]. In recent years, there has been growing research interest on prosthetic feet. Publications include review papers, original articles, conference proceedings, and case reports which have investigated the outcome measures of prosthetic feet [7,10,11,12,13,14,15], the effects of prosthetic feet on those with an LEA [11,12,13,16], design guidelines and principles [9,17,18], and the latest developments and trends [8,17,19] of prostheses for lower limb amputees.

Although the aforementioned studies shed light on prosthetic feet, they only focus on one specific field in prosthetic foot research, and a more comprehensive scientometric analysis of the prosthetic foot is lacking in the literature. Scientometrics is used to evaluate and analyze scientific research in a given subject or field [20]. Compared to descriptive literature reviews (e.g., conventional reviews), scientometrics has been proven to have the advantage of efficiently identifying critical issues and guiding future research work [20,21,22]. In this article, a scientometric analysis which includes a bibliometric analysis is carried out. Bibliometric analysis is used to quantitatively explore and identify the scientific structures and trends (e.g., countries, journals, publications, and institutions) of the target study via statistics and mathematics [20,21,23]. The relevant nodes and useful information can be extracted and identified by using this method [20]. Scientific mapping can effectively facilitate bibliometric visualization to provide new perspectives and insights, as well as suggest future research directions [20,23]. However, a scientometric analysis of prosthetic feet has not yet been performed in the literature.

The objective of this study was to comprehensively explore the research trends and hotspots from 2000 to 2022, as well as suggested future research directions related to prosthetic feet by utilizing the scientometric method. The statistical results of the keyword analysis were summarized and categorized, which had a direct influence on uncovering critical evidence and highlighted emerging trends in prosthetic foot research work.

## 2. Methods

### 2.1. Data Acquisition and Search Strategy

**Figure 1:**
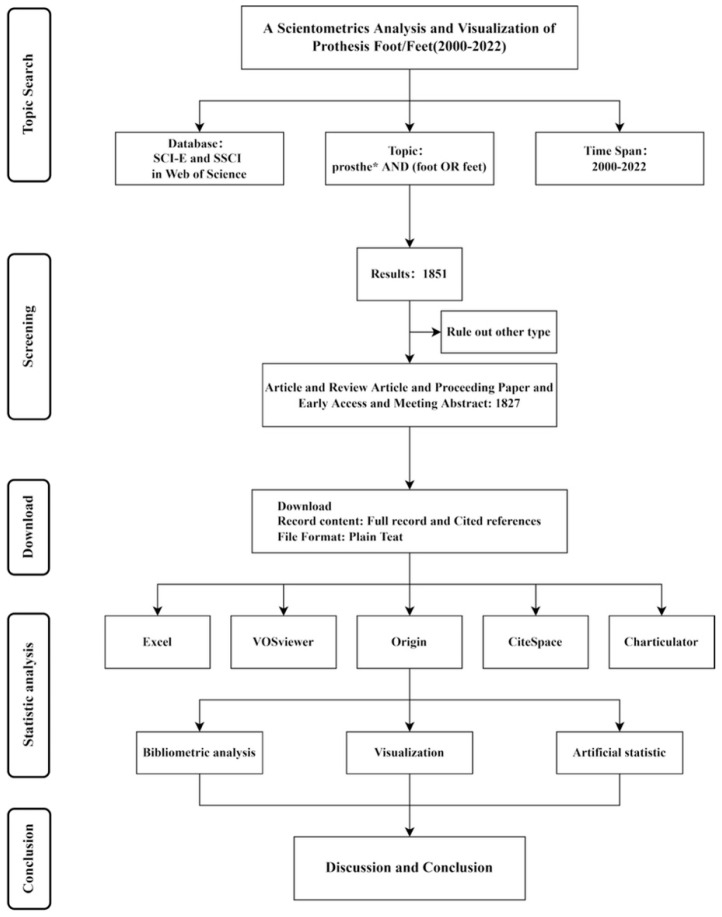
Flow chart of scientometric analysis. SCI-E, Science citation index expanded; SSCI, Social sciences citation index. * indicates one and more root words, referring to terms related to a prosthesis or prosthetic.

Two researchers developed the search strategy for screening key information to define keywords related to our topic and exclude unmeaningful words (e.g., population, and review), while another two experienced research librarians double checked this strategy to prevent researcher bias [24,25]. The search was conducted on 31 October 2022 in the Clarivate Analytics Web of Science (WoS) Core Collection. The WoS Core Collection database includes the Science Citation Index Expanded (SCI-E) and social sciences citation index (SSCI) which were systematically searched from 1 January 2000 to 31 October 2022. The WoS is a comprehensive and the most frequently used database for bibliometric research in previous studies [20,23]. The scientometric research process is shown in Figure 1.

We used “prosthe*” and “foot” or “feet” as the topical retrieval terms. Original articles, reviews with full manuscripts, conference proceedings, early access documents, and meeting abstracts were included. Other documents, including letters, corrections, editorial materials, and literature reviews with briefly summarized publications, consensus statements, and guidelines were excluded due to the lack of high-level scientific metrics in the field. The language was restricted to English. A total of 1851 articles were included for further analysis.

### 2.2. Data Extraction and Analysis

Microsoft Excel 2022 (Microsoft Corp., Redmond, WA, USA), VOSviewer (version 1.6.15), Origin, CiteSpace (version V), and Charticulator were selected for the bibliometric analysis with bibliometric information related to the publications, including country, institution, journal, references, keywords, and categories [26,27]. Some of the other bibliometric information includes the number of publications, impact factor, H-index, degree of centrality, strength of linkage, year of publication, and occurrence/citation bursts were extracted and analyzed. CiteSpace (6.1.R3) was applied to detect the keywords and references with citation bursts, while the citation bursts included strength and duration and indicated that a paper was highly cited in a certain period [28]. Centrality is based on the theory of social network analysis by counting the number of shortest paths between other nodes in interest [29]. The impact factor in this study is based on the Journal Citation Reports (2022). The parameters include time slices (2000–2022), types of nodes, and selection criteria (i.e., the 25 most cited or representative publications or keywords). VOSviewer was used to construct and visualize the bibliometric map.

## 3. Results

### 3.1. Publication Outputs

**Figure 2:**
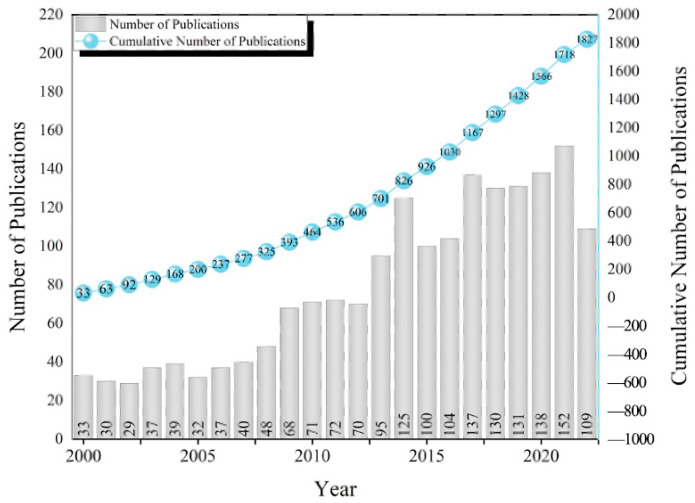
Related publications between 2000 and 2022.

A total of 1827 publications met the search criteria. Figure 2 shows the distribution of the publications by year between 1 January 2000 and 31 October 2022. The number of papers doubled between 2000 and 2008, while there was a stable increase between 2009 and 2012, and more related papers were published after 2013. However, there was a drop in 2015 but an increase in 2017 again, with a stable increase afterwards.

### 3.2. Distribution by Country and Institution

**Figure 3:**
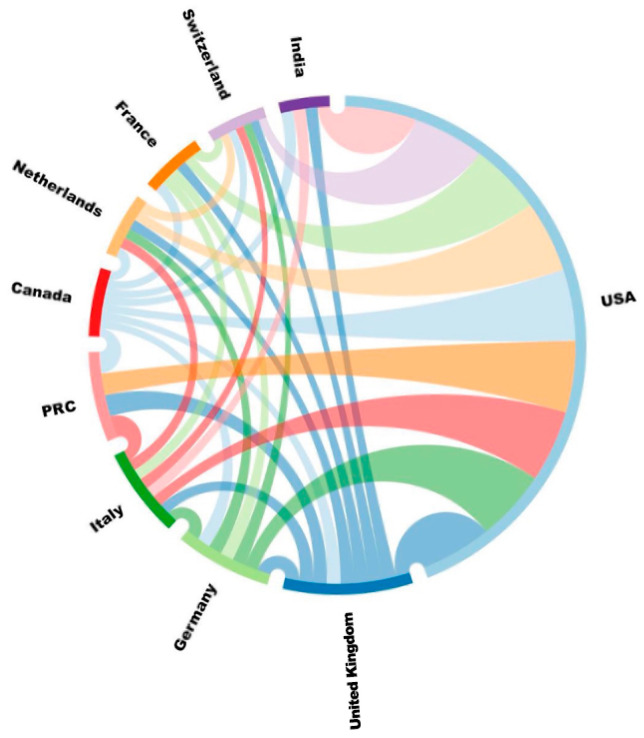
Top ten countries with the highest number of collaborative prosthetic foot publications. PRC denotes the People’s Republic of China.

The network analysis of the scientific collaborations was conducted by using Citespace software. Figure 3 shows the collaborative relationships based on country for the top 10 countries with the highest number of collaborative publications between 2000 and 2022. The highest number of publications is found for the United States (774 or 42.3%), followed by the United Kingdom, Germany, Italy, and China, with more than 100 publications (25.7%). The centrality of the United States also ranks first (0.51), followed by Italy (0.25), and the United Kingdom (0.22) (see Table 1).

Table 2 presents the top 10 institutions with the highest number of publications including centers, institutes, and universities. Northwestern University ranks first (84), followed by the University of Washington (60), and the University of Michigan. It can be observed that 9 of the top 10 institutions are in the United States.

### 3.3. Distribution by Journal

The number of publications found in the 15 journals with the most publications on prosthetic feet varies from 26 to 151 (average 53.7), accounting for 44.037% of the total number of publications (see Table 3). Among these journals, *Prosthetics and Orthotics International* published the most articles (151), followed by *Foot and Ankle International* (85), and the *Journal of Foot and Ankle Surgery* (67). Eight of the fifteen journals are located in the United States, five in the UK, and the remaining two in Ireland and Switzerland, respectively. Among these top 15 journals, the impact factor ranges from 1.277 to 6.558 (average 3.208). Seven journals have an impact factor > 3.000, which published 297 (or 16.3%) of these papers from 2000 to 2022. *The Journal of Bone and Joint Surgery American Volume* (6.558) has the highest impact factor among the top 15 journals, followed by the *Journal of NeuroEngineering and Rehabilitation* (5.208), and *IEEE Transactions on Neural Systems and Rehabilitation Engineering* (4.528).

### 3.4. Analysis of References

Prosthetic foot-related papers with the largest citation burst from 2000 to 2022 are listed in Table 4. In the first decade of 2000 to about 2010, the authors with the largest citation bursts (strength, time spam) were led by Gitter (1991) (14.04, 2000–2012), followed by Torburn (1990) (13.13, 2000–2013), and then Lehmann (1993) (11.47, 2000–2010). Shepherd (2017) (12.95, 2019–2022) has the largest citation burst at the end of 2022.

### 3.5. Analysis of Keywords

**Figure 4:**
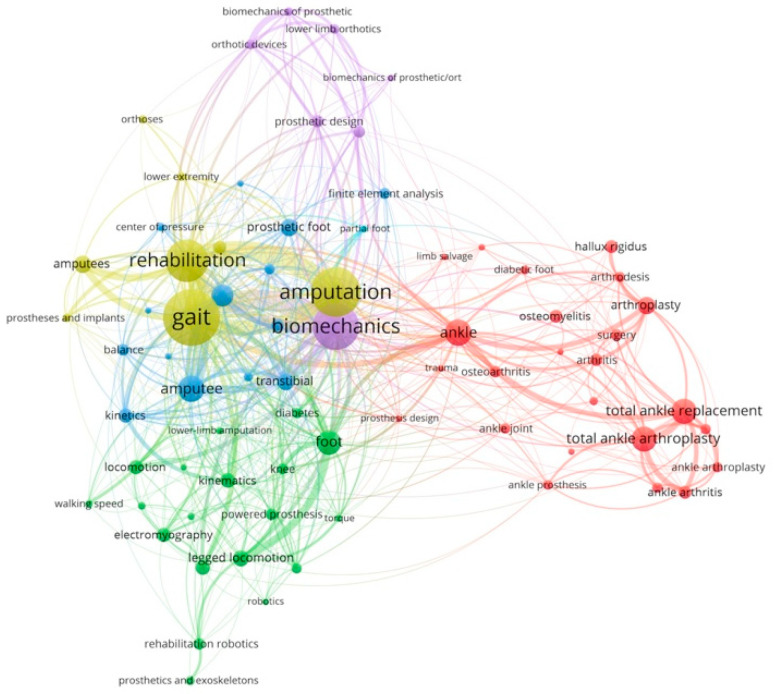
Keyword co-occurrence network. The colors indicate the keywords in different research fields; the size of the circle represents the importance of the keyword in this field; the link lines represent the connections among keywords.

**Figure 5:**
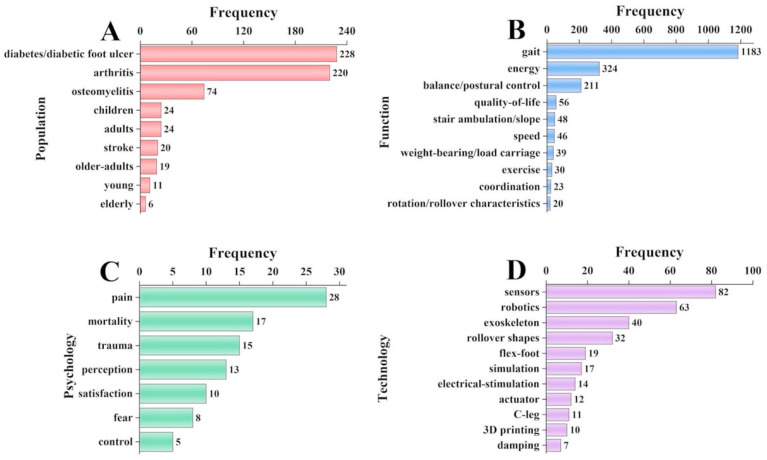
Results of manual analysis of keywords: (**A**) population, (**B**) function, (**C**) psychology, and (**D**) technology.

A keyword analysis was carried out to provide the hotspots and trends in prosthetic foot research work according to the number of occurrences. The keyword co-occurrence network is shown in Figure 4, where the node size and color represent the number of keywords and cluster, respectively. The different line colors show that the 2 keywords appear in an article. The largest node is gait. The keywords with citation bursts were further summarized, in which keywords were prioritized according to the number of citations during a given period of time (Figure 5). The keywords were categorized into four main areas: population, function, psychology, and technology. Figure 5A shows that diabetic patients are the main population studied in prosthetic foot research, followed by patients with rheumatoid arthritis, and individuals with osteomyelitis. The three main functions of the studied prosthetic feet include gait (e.g., movement, walking), energy (e.g., energy saving, energy storage), and balance of the user (Figure 5B). Pain is the main topic studied in the psychology realm (Figure 5C). Studies on prosthetic foot technologies focus the most on sensors, then robotics, and finally exoskeletons (Figure 5D).

## 4. Discussion

### 4.1. Main Findings

Scientometrics is used to comprehensively examine research work on prosthetic feet from 2000 to 2022. The major findings of the scientometric analysis are the top keywords with citation bursts for each category which systematically show the hotspots and future research directions regarding prosthetic feet, including population, function, psychology and technology.

### 4.2. Population

We found that the largest group of studies on LEA focuses on diabetes. Around 75% of LEAs are attributed to diabetes in the United States [30,31]. Diabetic foot is also the leading cause of amputation in Australia [32]. Previous hospital-based studies have reported that 53.3% of those with an LEA also have diabetes in Hong Kong [31,33]. Riandini et al. [34] reported that the highest amputation rates are found among those with diabetes (2008, 99.5/100,000; 2017, 95.0/100,000 people with diabetes), while amputation rates among non-diabetic Asians (i.e., Chinese, Malay, Indian, and others) are significantly lower (2008, 3.0/100,000; 2017, 2.1/100,00 individual without diabetes). Diabetic patients have 12 times higher risk of amputation when compared with non-diabetic individuals due to diabetic neuropathy [35,36,37]. Desveaux et al. [38] also found that Canadians with diabetes are 20 times more likely to be hospitalized for LEAs than non-diabetic Canadians. Diabetes is highly correlated with mortality after amputation [39]. The second largest group of individuals who might suffer from LEAs is those with arthritis-related diseases including osteoarthritis, knee osteoarthritis, and rheumatoid, ankle, and septic arthritis. Past studies have found that arthritis-related diseases are devastating, with a high potential for permanent disability and LEAs [40,41,42]. However, the results in this study show that the number of publications on LEAs among the elderly is still limited.

### 4.3. Function

In our study, the most frequently used keyword in prosthetic foot research work is gait. Gait is mostly found in studies on biomechanics, amputation, and rehabilitation (Figure 4). According to the burst frequency, gait (*n* = 1183, Figure 5B) is the major outcome to evaluate the functional performance of prosthetic feet in LEAs. Ghillebert et al. [13] found that 53% of the research publications in their study adopted biomechanical analysis as the outcome measure. Gard [11] used a quantitative gait analysis to fully describe the gait characteristics of those with an LEA and their rehabilitation progress. In quantitative gait analyses, measures such as spatiotemporal, kinematic, and kinetic measures have been addressed [11,13,15]. The spatiotemporal measures are collected across both space and time [43], while primarily incorporating gait parameters such as walking speed [44,45,46,47,48,49,50,51,52,53,54,55,56,57,58,59,60,61,62,63,64,65,66,67,68,69,70,71,72,73,74,75,76,77,78,79,80,81,82,83,84,85,86,87,88,89,90,91,92,93], cadence [46,53,56,75,76,94,95,96,97,98], stride length [49,53,56,64,75,96,99,100,101], step length [49,51,52,58,63,75,76,84,86,92,93,100,102,103,104,105,106,107], step width [64,70,75,84], stride time [104], stance time [46,49,51,52,53,54,56,58,62,64,65,66,69,70,71,76,80,84,85,87,90,91,92,93,95,96,98,99,104,107,108,109,110,111,112,113,114,115], and swing time [46,52,56,58,61,64,65,66,69,70,87,90,95,96,99,101,108,111,114]. The kinematics are measured by using motion capture systems that acquire data on ranges of motion [44,46,49,50,52,53,54,56,57,58,59,61,62,63,64,65,66,69,70,75,77,79,80,81,82,85,86,87,88,89,90,91,92,93,94,96,99,102,104,106,107,108,111,112,114,115,116,117,118,119,120,121,122,123,124,125,126,127,128,129,130], and center of motion [45,55,63,68,80,81,86,89,92,99,102,107,116,120,129,131]. The most frequently examined parameters of the kinetic measures include ground reaction forces [44,45,51,52,54,56,59,61,62,63,64,69,76,78,79,81,82,84,86,88,90,92,93,94,95,96,97,101,102,104,105,106,108,109,110,112,113,116,118,119,120,122,124,126,127,128,129,130,131,132], the center of pressure [52,55,84,89,105,107,109,110,112,119], power [46,50,52,55,56,57,58,59,63,71,77,78,79,80,81,83,85,87,88,89,92,93,94,98,99,100,102,104,106,108,109,114,115,116,117,120,121,123,124,125,126,127,128,129,130,131,133], and torque [46,50,52,55,56,57,58,59,63,71,77,78,79,80,81,83,85,87,88,89,92,94,98,99,100,104,106,108,109,114,115,116,117,120,121,123,124,125,126,127,128,130,131,133]. Most of the studies on gait have implemented experiments on a flat ground or during treadmill walking, with very few studies that use stairs [94,134,135] or assess walking on a slope [134]. Abnormal gait (e.g., externally rotated feet [136]) can be visually observed from the kinematic outcomes, which is useful for identifying the compensatory movement of those with an LEA during walking [11]. Sadeghi et al. [137] reported that reduced ankle power is found with the use of a prosthetic foot, while the increased power of the knee and hip joints is due to the residual limbs. The interface pressure [138] of the liner or socket is also a significant measurement that affects the mobility, wear comfort, and satisfaction of the amputee with their prosthetic foot.

Besides gait analyses, physiological measurements also include heart rate [19,47,95,100,103,134,139,140,141], oxygen consumption [57,58,67,72,73,74,81,86,103,123,129,131,134,139,140,142], and carbon dioxide production [57,58,74,81,103,123,129,131,134,139,140,142], which are used as indicators for evaluations of energy and metabolic costs with a prosthetic foot during walking. Previous studies also report measurements of energy expenditure in the form of questionnaires [47,49,50,53,54,57,58,60,61,62,67,68,70,75,81,83,95,98,119,120,123,134,140,142] like the visual analogue scale [54] and rate of perceived exertion [62,103,139]. The prosthetic foot is integrated into the involved lower extremities to facilitate the muscle–tendon units to interact with the skeletal system by generating, storing, dissipating, and transferring energy between segments [4,143,144], while stabilizing joints and controlling balance, thus saving more energy and allowing walking with less effort [4,145].

### 4.4. Psychology

Apart from gait and physical analyses, psychological variables associated with the use of a prosthetic foot (i.e., pain, mortality, trauma, perception, satisfaction, fear, and control) have also been measured to evaluate the rehabilitation outcomes for those with LEA. Psychological analyses are normally conducted by using a survey or carrying out an interview in a double-blinded study. Questionnaires like the Houghton scale [146,147], Locomotor Capabilities Index with a five-level ordinal scale [148,149,150], Mobility Section of the Prosthetic Evaluation Questionnaire [16,151,152], and prosthetic socket fit comfort score [16,153] are frequently used to understand the subjective feelings of lower limb amputees towards the prosthesis. Studies have found that over 50% of those with an LEA reported pain while using their prosthetic foot [154,155,156]. Pain and mental fear are high-risk factors for falls while walking [152], increasing mortality [39]. Singh and Prasad [39] found a mortality rate of 33% among the lower limb amputees in their study after three years of follow-up, which is significantly related to the absence of a prosthesis. More than half, or 57%, of those with an LEA reported dissatisfaction with the wear comfort of their prosthetic foot [155,156]. Their perception (e.g., wear comfort, trust, and security) of their prosthetic foot is linearly related to their physical activities [157], while also influencing their psychological well-being. They experience fear, anxiety, depression, self-esteem, and lack of life satisfaction [158]. Bunce and Breakey [158] found that 71% of their study participants with an LEA had experienced some form of trauma. Psychological factors affect how they control their prosthesis [152]. However, higher self-efficacy, family, and social supports increase the physical activity of those with an LEA [157].

### 4.5. Technology

To enhance the physical abilities and psychological resilience of those with an LEA and who use a prosthesis during different physical activities, prosthetic foot technologies have undergone a substantial transformation over recent decades. The rapid development of sensory technologies can be observed from keyword searches. Sensors like ActiGraph (Pensacola, FL, USA) [48], ActivPALTM (PAL Technologies, Glasgow, Scotland) [157,159], FitBit One (Fitbit, CA, USA) [160], StepWatchTM (Modus Health, Washington, DC, USA) [7,57,60,62,86,98], Power Walker EX (Yamasa, Tokyo, Japan) [47], and inertial measurement units (i.e., ActiGraph GT9X Link, and Acti-Graph, Pensacola, FL, USA) [48], which are widely used to monitor and collect data of steps walked per day, walking speed, and energy consumption, help to provide a better understanding of the rehabilitation progress of lower extremity amputees and their sedentary lifestyle. Apart from the sensory systems applied to collect mobility data, more and more recent technology-related work on robotic prostheses provides biological configuration information of the residual muscles [161], while considering the neural interface for prosthesis control [162]. Residual muscles can still be activated by the brain and spinal cord after amputation surgery [121,163], as evidenced by researchers who have obtained electromyography (EMG) signals from the residual muscles (e.g., adductor magnus, gluteus maximus, gluteus medius, hamstring, quadriceps, soleus, and tibialis) as the neural inputs to recognize different locomotion modes [164,165,166]. However, controlling EMG-based robotic prostheses is still limited by the accuracy and quality of the EMG signals [161]. Meanwhile, recent innovations in embedded computing systems and sensors have improved the functions of powered exoskeleton prostheses. Kim and Oh [167] developed a prosthesis by using a magnetorheological damper as a torque actuator, which is adaptable to different walking speeds. Flex-Foot, a foot prosthesis, has been proven to provide greater power absorption during early midstance, and a higher plantarflexion moment and power generation in the late stance, thus facilitating energy storage in midstance, and energy return in the late stance [168]. Ottobock C-Leg^®^ with a microprocessor knee can adapt to a wide range of mobilities (e.g., descending stairs, uneven terrain, or backward walking) [169].

To allow lower extremity amputees to walk more safely, naturally, and with ease during different locomotion modes, simulation technologies are used to assist with the development of prosthetic feet. Xu et al. [170] used kinetic modeling of control algorithms (i.e., Lagrange’s second kinetic equation, the governing equation, and constraint equation) to develop a lower limb prosthesis which can imitate the center of rotation of a knee joint accurately. Kim and Oh [167] developed a leg simulator to produce thigh movements in the horizontal, vertical, and rotational directions by modeling, so that dynamic data could be applied to develop prostheses for those with an LEA. In addition, 3D printing with composite fibers (e.g., carbon and glass fiber filaments), nylon, or metals is often used in clinical trials due to the reduction in the production time of prostheses, minimization of the costs, while improving the wear comfort to optimize prostheses for those with an LEA [171].

## 5. Conclusions

Scientometrics is applied to analyze studies in the literature from the SCI-E and SSCI databases. The findings of this study reveal the hotspots, key research directions, countries, institutions, and journals that focused on prosthetic feet from 2000 to 2022. Through an analysis of the keywords, we find that elderly diabetics are the largest group of lower extremity amputees, but research on prosthetic feet among the elderly who suffer from an LEA is still limited. Secondly, studies that focus on both physical and psychological parameters should be considered to evaluate the functions of prostheses with modern technologies. These findings not only show the current research hotspots but also reveal further research work for researchers and clinicians. The limitation is that the data for this study were obtained from the SCI-E and SSCI databases in the WoS from 2000 to 2022. While the top 25 articles with the highest citation bursts represent highly influential work in the literature for two decades, the current study msay not have captured all of the important articles in this field.

## Figures and Tables

**Table 1:** Statistics on top 10 countries with highest number of collaborative publications.

Number of Publications	Centrality	Country
774	0.51	USA
150	0.22	UNITED KINGDOM
112	0.06	GERMANY
104	0.25	ITALY
104	0.05	PRC
79	0.15	CANADA
74	0.07	THE NETHERLANDS
73	0.10	FRANCE
69	0.05	SWITZERLAND
59	0.01	INDIA

PRC denotes the People’s Republic of China; USA denotes the United States of America.

**Table 2:** Top 10 institutions with the largest number of published articles on prosthetic feet.

Number of Publications	Centrality	Name of Institution
84	0.08	Northwestern University
60	0.05	University of Washington
31	0.01	University of Michigan
23	0.03	Brooke Army Medical Center
22	0.01	Duke University
20	0.00	The University of Texas at Austin
18	0.02	Massachusetts Institute of Technology
17	0.03	Georgia Institute of Technology
17	0.00	Vrije Universiteit Brussel
16	0.01	Jesse Brown VA Medical Center

**Table 3:** Top 15 journals with the most publications on prosthetic feet.

Rank	Journal	Number of Publications	% of Total Number of Publications	Country	Impact Factor (2022)	H-Index (2022)
1	Prosthetics and Orthotics International	151	8.26	UK	1.672	55
2	Foot and Ankle International	85	4.65	USA	3.569	114
3	Journal of Foot and Ankle Surgery	67	3.67	USA	1.345	70
4	Gait and Posture	62	3.39	Ireland	2.746	156
s	Journal of Biomechanics	58	3.17	UK	2.789	208
6	Journal of Rehabilitation Research and Development	56	3.06	USA	1.277	108
7	IEEE Transactions on Neural Systems and Rehabilitation Engineering	53	2.90	USA	4.528	148
8	Clinical Biomechanics	48	2.63	UK	2.034	135
9	PLoS ONE	40	2.19	USA	3.752	367
10	Foot and Ankle Surgery	38	2.08	UK	2.840	43
11	Journal of NeuroEngineering and Rehabilitation	32	1.75	UK	5.208	102
12	Journal of Bone and Joint Surgery American Volume	31	1.70	USA	6.558	274
13	Archives of Physical Medicine and Rehabilitation	30	1.64	USA	4.060	197
14	Journal of Biomechanical Engineering-Transactions of the ASME	28	1.53	USA	1.899	131
15	Sensors	26	1.42	Switzerland	3.847	196

**Table 4:** References with the largest citation bursts between 2000 and 2022.

References	Year	Strength	Begin	End	2000–2022
GITTER A, 1991, AM J PHYS MED REHAB, V70, P142, https://doi.org/10.1097/00002060-199106000-00006	1991	14.04	2000	2012	▃▃▃▃▃▃▃▃▃▃▃▃▃ ▂▂▂▂▂▂▂▂▂▂
TORBURN L, 1990, JOURNAL OF REHABILITATION RESEARCH AND DEVELOPMENT, V27, P369, https://doi.org/10.1682/JRRD.1990.10.0369	1990	13.13	2000	2013	▃▃▃▃▃▃▃▃▃▃▃▃▃▃ ▂▂▂▂▂▂▂▂▂
LEHMANN JF, 1993, ARCH PHYS MED REHAB, V74, P1225, https://doi.org/10.1016/S0003-9993(23)00022-9	1993	11.47	2000	2010	▃▃▃▃▃▃▃▃▃▃▃ ▂▂▂▂▂▂▂▂▂▂▂▂
LEHMANN JF, 1993, ARCH PHYS MED REHAB, V74, P853, https://doi.org/10.1016/0003-9993(93)90013-Z	1993	9.95	2000	2006	▃▃▃▃▃▃▃ ▂▂▂▂▂▂▂▂▂▂▂▂▂▂▂▂
POWERS CM, 1994, ARCH PHYS MED REHAB, V75, P825, https://doi.org/10.1016/0003-9993(94)90146-5	1994	9.86	2000	2012	▃▃▃▃▃▃▃▃▃▃▃▃▃ ▂▂▂▂▂▂▂▂▂▂
SNYDER RD, 1995, J REHABIL RES DEV, V32, P309	1995	9.67	2000	2010	▃▃▃▃▃▃▃▃▃▃▃ ▂▂▂▂▂▂▂▂▂▂▂▂
ARYA AP, 1995, PROSTHET ORTHOT INT, V19, P37, https://doi.org/10.3109/03093649509078230	1995	7.51	2000	2008	▃▃▃▃▃▃▃▃▃ ▂▂▂▂▂▂▂▂▂▂▂▂▂▂
POSTEMA K, 1997, PROSTHET ORTHOT INT, V21, P28, https://doi.org/10.3109/03093649709164527	1997	7.37	2000	2004	▃▃▃▃▃ ▂▂▂▂▂▂▂▂▂▂▂▂▂▂▂▂▂▂
KADABA MP, 1990, J ORTHOP RES, V8, P383, https://doi.org/10.1002/jor.1100080310	1990	8.50	2004	2010	▂▂▂▂ ▃▃▃▃▃▃▃ ▂▂▂▂▂▂▂▂▂▂▂▂
ANDERSON T, 2003, J BONE JOINT SURG AM, V85A, P1321, https://doi.org/10.2106/00004623-200307000-00019	2003	7.66	2006	2014	▂▂▂ ▂▂▂ ▃▃▃▃▃▃▃▃▃ ▂▂▂▂▂▂▂▂
HANSEN AH, 2006, PROSTHET ORTHOT INT, V30, P286, https://doi.org/10.1080/03093640600816982	2006	10.62	2008	2012	▂▂▂▂▂▂ ▂▂ ▃▃▃▃▃ ▂▂▂▂▂▂▂▂▂▂
BONNIN M, 2004, CLIN ORTHOP RELAT R, V0, PP6, https://doi.org/10.1097/01.blo.0000132407.75881.a0	2004	8.27	2009	2015	▂▂▂▂ ▂▂▂▂▂ ▃▃▃▃▃▃▃ ▂▂▂▂▂▂▂
SILVERMAN AK, 2008, GAIT POSTURE, V28, P602, https://doi.org/10.1016/j.gaitpost.2008.04.005	2008	9.51	2011	2012	▂▂▂▂▂▂▂▂ ▂▂▂ ▃▃ ▂▂▂▂▂▂▂▂▂▂
VALDERRABANO V, 2004, CLIN ORTHOP RELAT R, V0, PP47, https://doi.org/10.1097/01.blo.0000132245.18548.09	2004	8.03	2011	2015	▂▂▂▂ ▂▂▂▂▂▂▂ ▃▃▃▃▃ ▂▂▂▂▂▂▂
HITT J, 2009, IND ROBOT, V36, P441, https://doi.org/10.1108/01439910910980169	2009	9.92	2013	2017	▂▂▂▂▂▂▂▂▂ ▂▂▂▂ ▃▃▃▃▃ ▂▂▂▂▂
WINTER DA, 1983, CLIN ORTHOP RELAT R, V0, P147	1983	8.07	2013	2018	▂▂▂▂▂▂▂▂▂▂▂▂▂ ▃▃▃▃▃▃ ▂▂▂▂
HSU MJ, 2006, ARCH PHYS MED REHAB, V87, P123, https://doi.org/10.1016/j.apmr.2005.07.310	2006	7.93	2013	2018	▂▂▂▂▂▂ ▂▂▂▂▂▂▂ ▃▃▃▃▃▃ ▂▂▂▂
FEY NP, 2011, CLIN BIOMECH, V26, P1025, https://doi.org/10.1016/j.clinbiomech.2011.06.007	2011	7.80	2016	2020	▂▂▂▂▂▂▂▂▂▂▂ ▂▂▂▂▂ ▃▃▃▃▃ ▂▂
QUESADA RE, 2016, J BIOMECH, V49, P3452, https://doi.org/10.1016/j.jbiomech.2016.09.015	2016	8.64	2017	2022	▂▂▂▂▂▂▂▂▂▂▂▂▂▂▂▂ ▂ ▃▃▃▃▃▃
ADAMCZYK PG, 2017, HUM MOVEMENT SCI, V54, P154, https://doi.org/10.1016/j.humov.2017.04.005	2017	7.50	2018	2022	▂▂▂▂▂▂▂▂▂▂▂▂▂▂▂▂▂ ▂ ▃▃▃▃▃
SHEPHERD MK, 2017, IEEE T NEUR SYS REH, V25, P2375, https://doi.org/10.1109/TNSRE.2017.2750113	2017	12.95	2019	2022	▂▂▂▂▂▂▂▂▂▂▂▂▂▂▂▂▂ ▂▂ ▃▃▃▃
HUANG H, 2011, IEEE T BIO-MED ENG, V58, P2867, https://doi.org/10.1109/TBME.2011.2161671	2011	9.71	2019	2022	▂▂▂▂▂▂▂▂▂▂▂ ▂▂▂▂▂▂▂▂ ▃▃▃▃
TUCKER MR, 2015, J NEUROENG REHABIL, V12, P0, https://doi.org/10.1186/1743-0003-12-1	2015	8.32	2019	2022	▂▂▂▂▂▂▂▂▂▂▂▂▂▂▂ ▂▂▂▂ ▃▃▃▃
GLANZER EM, 2018, IEEE T NEUR SYS REH, V26, P2351, https://doi.org/10.1109/TNSRE.2018.2877962	2018	9.81	2020	2022	▂▂▂▂▂▂▂▂▂▂▂▂▂▂▂▂▂▂ ▂▂ ▃▃▃
LAWSON BE, 2014, IEEE ROBOT AUTOM MAG, V21, P70, https://doi.org/10.1109/MRA.2014.2360303	2014	8.44	2020	2022	▂▂▂▂▂▂▂▂▂▂▂▂▂▂ ▂▂▂▂▂▂ ▃▃▃

Notes: Red bars indicate a high surge of citations; green bars a small surge of citations.

## Data Availability

Data are available upon reasonable request. If you want to request the data from this study, please contact the corresponding author, Kit-lun Yick.
